# Metabolomics Analysis of Amniotic Fluid in Euploid Foetuses with Thickened Nuchal Translucency by Gas Chromatography-Mass Spectrometry

**DOI:** 10.3390/life11090913

**Published:** 2021-09-02

**Authors:** Federica Murgia, Giovanni Monni, Valentina Corda, Aran J. Hendren, Giulia Paci, Alba Piras, Rosa M. Ibba, Luigi Atzori

**Affiliations:** 1 Clinical Metabolomics Unit, Department of Biomedical Sciences, University of Cagliari, 09042 Monserrato, Italy; giuliapeace94@gmail.com (G.P.); latzori@unica.it (L.A.); 2 Department of Prenatal and Preimplantation Genetic Diagnosis and Fetal Therapy, Ospedale Pediatrico Microcitemico A.Cao, 09121 Cagliari, Italy; cordavale@hotmail.it (V.C.); albapirasmed@gmail.com (A.P.); rosamariaibba@gmail.com (R.M.I.); 3 Faculty of Health and Medical Sciences, University of Surrey, Guildford GU2 7XH, UK; ah01169@surrey.ac.uk

**Keywords:** prenatal diagnosis, ultrasound foetal nuchal translucency, metabolomics, amniotic fluid, gas chromatography mass spectrometry, energetic pathways

## Abstract

Persistence of a fetal thickened nuchal translucency (NT), one of the most sensitive and specific individual markers of fetal disorders, is strongly correlated with the possibility of a genetic syndrome, congenital infections, or other malformations. Thickened NT can also be found in normal pregnancies. Several of its pathophysiological aspects still remain unexplained. Metabolomics could offer a fresh opportunity to explore maternal–foetal metabolism in an effort to explain its physiological and pathological mechanisms. For this prospective case-control pilot study, thirty-nine samples of amniotic fluids were collected, divisible into 12 euploid foetuses with an enlarged nuchal translucency (>NT) and 27 controls (C). Samples were analyzed using gas chromatography mass spectrometry. Multivariate and univariate statistical analyses were performed to find a specific metabolic pattern of >NT class. The correlation between the metabolic profile and clinical parameters was evaluated (NT showed an R^2^ = 0.75, foetal crown-rump length showed R^2^ = 0.65, pregnancy associated plasma protein-A showed R^2^ = 0.60). Nine metabolites significantly differing between >NT foetuses and C were detected: 2-hydroxybutyric acid, 3-hydroxybutyric, 1,5 Anydro-Sorbitol, cholesterol, erythronic acid, fructose, malic acid, threitol, and threonine, which were linked to altered pathways involved in altered energetic pathways. Through the metabolomics approach, it was possible to identify a specific metabolic fingerprint of the fetuses with >NT.

## 1. Introduction

Genetic screening and invasive prenatal diagnosis paradigms have been profoundly modified in the last 30 years owing to the introduction of innovative technologies and improved operator skills [1]. Novel approaches to ultrasound and biochemical screening, the detection of foetal abnormalities, reducing foetal loss risk following invasive prenatal procedures, and increasing the accuracy of molecular analysis are in continuous development [1,2,3].

Among contemporary developments is the rapid diffusion of non-invasive prenatal screening (NIPS), using foetal cell-free DNA (cfDNA) obtained from maternal blood, as a diagnostic technique for aneuploidies [2]. However, the scientific community must carefully consider the impact that NIPS may have on more well-established methods of prenatal screening and diagnosis [4,5].

Despite recent progress, first trimester combined screening, which is performed between 11 and 13^+6^ weeks of gestation by calculating the risk of aneuploidies using maternal age, ultrasound foetal nuchal translucency (NT) thickness, foetal heart rate (FHR), maternal serum-free ß-human chorionic gonadotropin (ß-hCG), and pregnancy associated plasma protein-A (PAPP-A), is still of paramount importance for risk assessment in pregnancy [6].

NT is a fluid-filled space in the posterior neck of the foetus, between the skin and the soft tissue at the level of the cervical spine, as visualized by ultrasound [7]. NT normally increases with the foetal crown-rump length (CRL) [8]. The thickened NT represents one of the most sensitive and specific individual markers of aneuploidy, structural defects, biometric discrepancies, and deviations from normal anatomy [9].

Persistence of a thickened NT is strongly correlated with the possibility of a genetic syndrome, congenital infections, or other malformations such as congenital heart disease or delayed development of the lymphatic system [10,11]. Nevertheless, a thickened NT can also be found in normal pregnancies [11]. Although this phenomenon has already been widely analysed in the literature, several of its pathophysiological aspects still remain unexplained. Metabolomics could offer a fresh opportunity to explore maternal–foetal metabolism in an effort to explain its physiological and pathological mechanisms [12].

Several metabolomics studies have been performed in the prenatal period on chorionic villi and amniotic fluid [13,14,15,16]; regarding the presence of a thickened NT, metabolomics could aid in clarifying the mechanisms of excessive accretion of fluids in foetuses, improving the performance of combined screening and honing an increasingly tailored diagnostic approach.

In a recent study, our research group analysed the metabolic composition of amniotic fluids of foetuses with enlarged NT with a nuclear magnetic resonance approach [17], obtaining interesting results. The aim of the current study is to ratify, challenge, or augment our previous results, using a complementary analytical approach such as gas chromatography-mass spectrometry (GC-MS).

GC-MS can provide information about tissue metabolites, such as lipids, amino acids, and high-energy metabolites [18,19], providing a “snapshot” of the metabolic profile under different conditions [20,21]. Quantification of metabolites allows identification of altered metabolic pathways through the use of pattern recognition techniques [22].

We performed a metabolomic study recruiting euploid fetuses with >NT, including the minimum number of subjects per group to be considered for pilot studies [23], with the aim to explore the metabolic profile of the amniotic fluid, in order to investigate the underpinning pathological or physiological mechanisms.

## 2. Patients and Methods

### 2.1. Patients

This prospective study was conducted in the Department of Obstetrics and Gynecology in the Microcitemico Paediatric “A. Cao” Hospital of Cagliari.

Amniotic fluids were collected from women who underwent amniocentesis due to an increased risk of aneuploidy. All patients had first trimester combined screening for aneuploidy via ultrasound measurement of foetal NT and CRL in combination with biochemical markers, such as PAPP-A and free β-hCG. All amniocentesis procedures were performed between 15 and 18 weeks of gestation with a free-hand transabdominal technique. Written consent was obtained from all participating women and the study was approved by the Institutional Review Board of Microcitemico Hospital. Patient demographic characteristics and ultrasonographic data (ethnic group, age, CRL, NT value, free β-hCG, and PAPP-A) were collected; all information is reported in Table 1 and the statistical details of the comparisons between the two classes of patients are reported in the Supplementary Materials (Figure S1).

All samples were kept at −80 °C until use. All pregnancies were followed longitudinally; the corresponding foetal, maternal, and neonatal data were collected and retained in a database.

### 2.2. Sample Preparation and Data Analysis

Samples were extracted as follows: 200 µL of each sample was mixed with 400 µL of acetone (containing succinic acid-2,2,3,3-d4 as an internal standard (Sigma-Aldrich, St. Louis, MO, USA)), vortexed, and then centrifuged at 10,000 rpm for 10 min at 4 °C. The supernatant was transferred to an Eppendorf tube and dried under vacuum overnight with an Eppendorf^TM^ Concentrator Plus. Dried extracts were derivatised with 50 μL of methoxyamine dissolved in pyridine (10 mg/mL, Sigma-Aldrich, St. Louis, MO, USA) at 70 °C. After 1 h, 100 μL of N-Methyl-N-(trimethylsilyl)-trifluoroacetamide (MSTFA, Sigma-Aldrich, St. Louis, MO, USA) was added and left at room temperature for one hour. Successively, samples were diluted in 100 μL of hexane (Sigma-Aldrich, St. Louis, MO, USA). The GC-MS analysis and the data processing were performed as previously described [24].

### 2.3. Statistical Analysis

A multivariate statistical analysis was performed using SIMCA-P software [25] (ver. 15.0, Sartorius Stedim Biotech, Umea, Sweden). Initially, variables (metabolites) were UV scaled and then principal component analysis (PCA) was applied, with the aim to explore the sample distributions without classification, and to identify potential strong outliers through the application of the Hotelling’s T^2^ tests. Subsequently, a partial least square discriminant analysis (PLS-DA) was performed. This type of model allows to observe, if present, differences between samples assigned to different classes [26]. The variance and the predictive ability (R^2^X, R^2^Y, Q^2^) were evaluated to establish the suitability of the models. In addition, a permutation test (n = 400) was performed to validate the models [27]. The scores from the PLS-DA model were subjected to a CV-ANOVA (analysis of variance testing of cross-validated predictive residuals) test to establish the significance of the separation between the two classes of patients (*p* < 0.05).

To study a possible linear relationship between a matrix Y (dependent variable, foetal nuchal thickness, PAPP-A, and free β-hCG) and a matrix X (predictor variables, e.g., metabolites), PLS models were carried out [28].

The most significant variables were extracted from the PLS-DA model’s loading plot. The variables’ influence on projection (VIP) value was also evaluated for the selection of the discriminant metabolites. In particular, when the value was >1, the metabolite was considered relevant for explaining Y (assignment to the two classes, controls and >NT). To verify the significance of the metabolites deemed noteworthy by multivariate statistical analysis, U-Mann Whitney tests were performed. GraphPad Prism software (version 7.01, GraphPad Software, Inc., San Diego, CA, USA) was used to conduct this univariate statistical analysis.

### 2.4. Pathways’ Analysis 

Metabolic pathways were generated using MetaboAnalyst 5.0 [29]. This approach allows to obtain a comprehensive metabolomic data interpretation, correlating metabolite alterations in specific pathways. In particular, this tool combines the results from pathway enrichment analysis with pathway topology analysis to help researchers identify the most relevant pathways involved in the conditions under study.

## 3. Results

**Figure 1:**
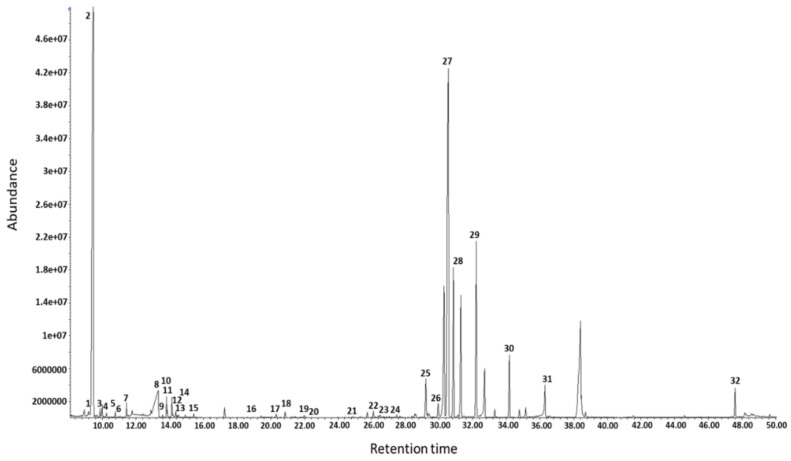
Main compounds identified by GC-MS: 1. Pyruvic acid, 2. lactic acid, 3. valine, 4. alanine, 5. 2-hydroxybutyric acid, 6. 3-hydroxybutyric acid, 7. isoleucine, 8. urea, 9. uerine, 10. glycerol, 11. threonine, 12. glycine, 13. succinic acid, 14. glyceric acid, 15. fumaric acid, 16. malic acid, 17. threitol, 18. erythronic acid, 19. threonic acid, 20. α-ketoglutaric acid, 21. xylulose, 22. xylitol, 23. ribitol, 24. ribonic acid, 25. 1,5-anydrosorbitol, 26. fructose, 27. glucose, 28. sorbitol, 29. palmitic acid, 30. myoinositol, 31. stearic acid, 32. cholesterol.

Thirty-nine samples of amniotic fluid were analysed with GC-MS, divisible into 12 patients with an enlarged nuchal translucency (>NT) and 27 controls (C). A total of 32 metabolites, including organic acids, amino acids, fatty acids, and sugars, were identified in amniotic fluid samples (Figure 1).

**Figure 2:**
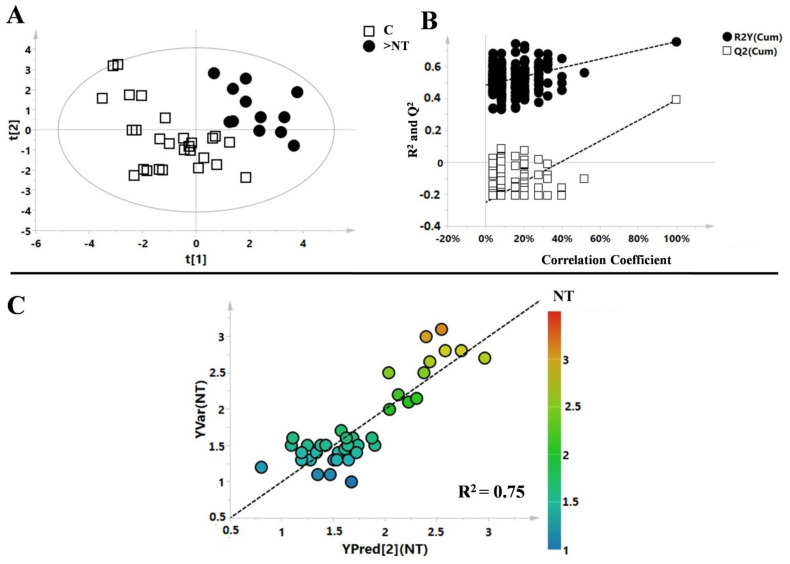
(**A**) PLS-DA model of enlarged nuchal translucency patients (>NT, black circle) and controls (C, white box) with the respective permutation test (**B**). (**C**) PLS correlation analysis of the metabolic profile with the values of the NT. Each point represents a subject enrolled in the study, while the points’ colors pertain to the value of the clinical parameter under consideration.

The data were organised into a matrix, which then underwent multivariate statistical analysis. Firstly, the PCA model was evaluated, and the Hotelling’s T2 test identified the presence of one outlier affecting the model (belonging to the control class, Figure S2). The supervised model obtained with the PLS-DA analysis showed robust statistical parameters: R^2^X = 0.2, R^2^Y = 0.76, Q^2^ = 0.4, *p* < 0.002 (Figure 2A).

**Figure 3:**
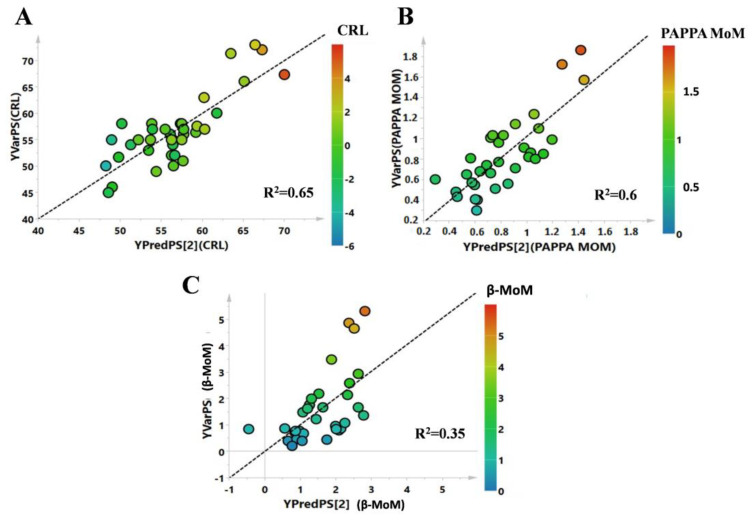
PLS correlation models of the metabolic profile with the statistical parameters (**A**) CRL, (**B**) PAPPA MoM, and (**C**) β-MoM. Each point represents a subject enrolled in the study, while their colors depend on the value of the clinical parameters considered.

The model was then validated with a permutation test (R^2^ intercept = 0.48; Q^2^ intercept = −0.27) (Figure 2B). Subsequently, analysis of correlations between the complete metabolic profile and NT in patients with abnormal NT values was completed via partial least squares (PLS) regression analysis (Figure 2C). The correlation between the complete metabolic profile and NT measured in the same patients showed an R^2^ = 0.75, *p* = 0.002. PLS correlation analysis was also performed to evaluate correlations between the metabolic profile of patients and others clinical parameters, such as CRL, PAPP-A MoM, and free β-hCG MoM. CRL and PAPP-A MoM showed R^2^ values of 0.65 and 0.6, respectively, while free β-hCG MoM showed no correlation (R^2^ equal to 0.35, Figure 3A, B, and C, respectively). 

**Figure 4:**
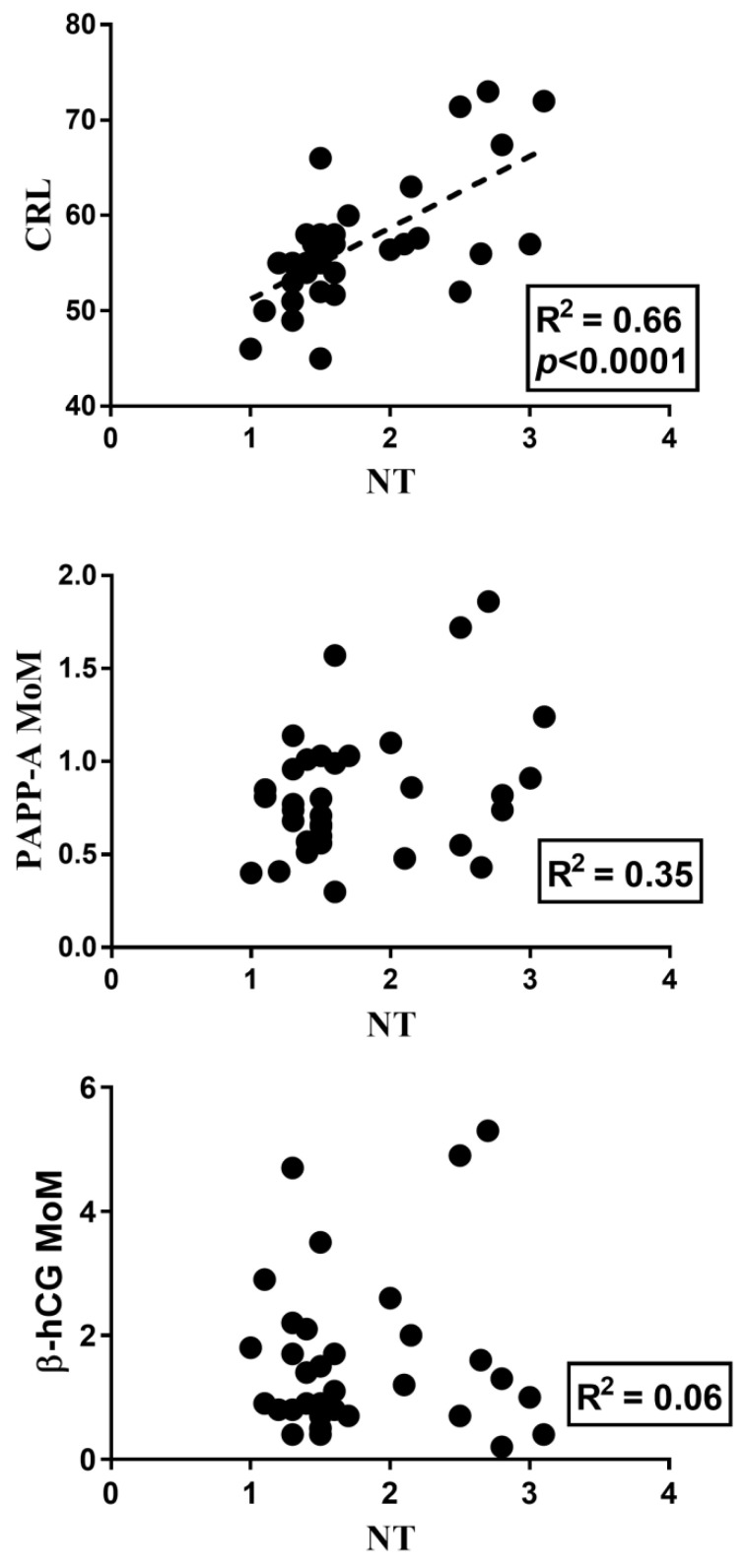
Pearson correlation of the clinical parameters NT, CRL, PAPP-A MoM, and free β-hCG MoM. The values of the NT strongly correlated with the CRL (R^2^ = 0.7, *p* < 0.0001), while no correlations were observed for both PAPP-A MoM and free β-hCG MoM (R^2^ = 0.32 and 0.06, respectively).

Moreover, the clinical parameters were correlated between them through the Pearson correlation. The values of the NT strongly correlated with the CRL (R^2^ = 0.7, *p* < 0.0001), while no correlations were observed for both PAPP-A MoM and free β-hCG MoM (R^2^ = 0.32 and 0.06, respectively, Figure 4).

Through analysis of the loadings plot and the VIP values of the PLS-DA model, it was possible to identify the variables responsible for the class separation. Discriminant metabolites were highlighted and then a U-Mann Whitney test was carried out to find significant differences in concentrations.

**Figure 5:**
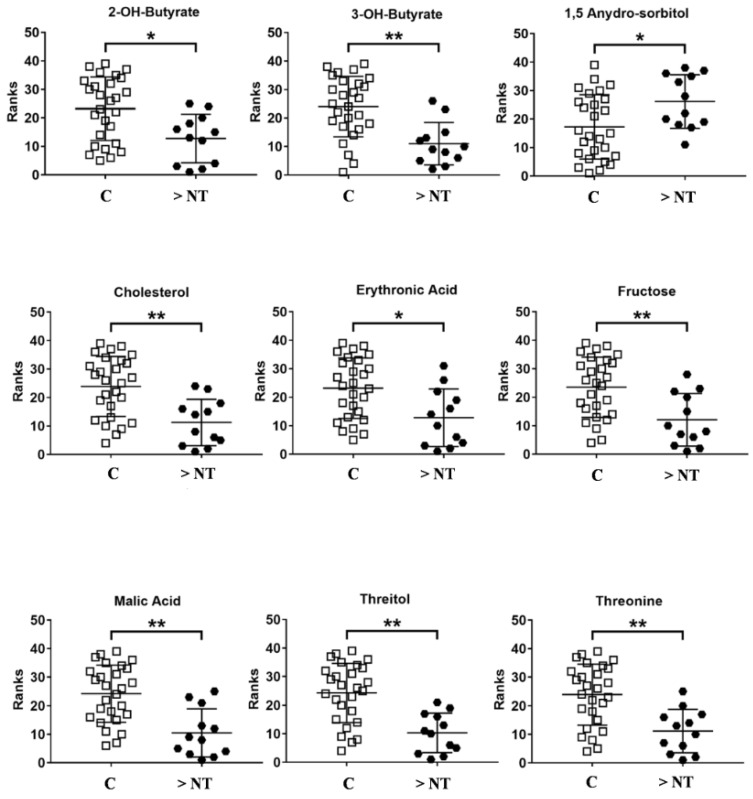
Graphs of the metabolites with *p*-values < 0.05 after U-Mann Whitney test and Holm–Bonferroni correction.* = *p* < 0.05; ** = *p* < 0.001.

Nine metabolites significantly differing between patients with enlarged NT and healthy controls were detected and deemed to be responsible for the separation of the two groups: 2-hydroxybutyric acid, 3-hydroxybutyric, cholesterol, erythronic acid, fructose, malic acid, threitol, and threonine were found to be decreased in the >NT group, while 1,5 Anydro-Sorbitol was found to be increased in the same group (Figure 5).

All statistical information for each metabolite is reported in Table 2. In particular, trend, *p*-value, adjusted *p*-value, and the parameters of the respective ROC curves are listed.

Metabolites showing AUC > 0.8 (3-OH butyrate, cholesterol, malic acid, threitol, and threonine) were combined to build a total ROC curve with parameters as follows: AUC = 0.91, standard error = 0.04, CI = 0.82–0.99, *p*-value < 0.0001.

**Figure 6:**
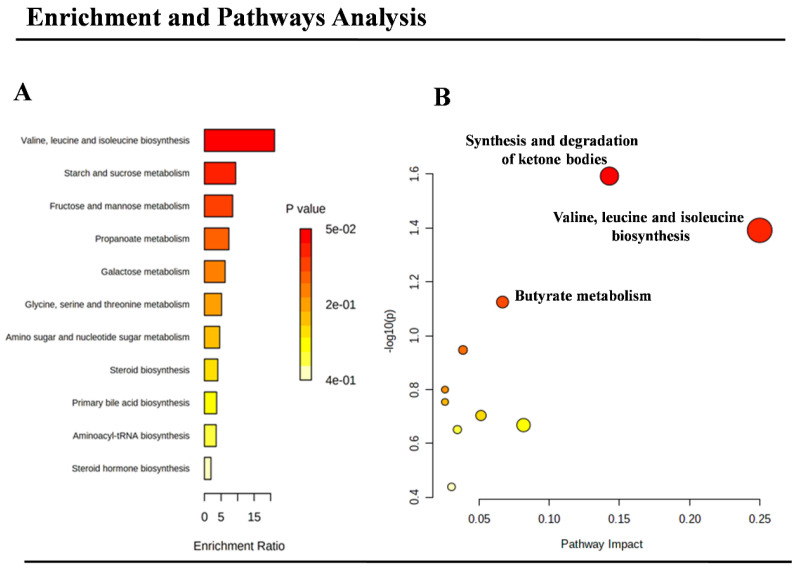
The metabolic pathways most altered in foetuses with enlarged nuchal translucency. Pathways (**A**) and enrichment (**B**) analysis were conducted: synthesis and degradation of ketone bodies, butyrate metabolism, valine, leucine, and isoleucine biosynthesis, and the metabolism of other carbohydrates, were the most altered.

The significant metabolites identified by the multivariate analysis were then used to identify the most significant metabolic pathways involved in >NT foetuses. To meet this aim, Metaboanalyst 5.0 was employed to carry out both enrichment and pathways’ analysis. These dual approaches identified the synthesis and degradation of ketone bodies, butyrate metabolism, valine, leucine, and isoleucine biosynthesis, and the metabolism of other carbohydrates, as significant in the pathogenesis of >NT (Figure 6A,B).

## 4. Discussion

“Omics sciences” aim at the comprehensive and quantitative analysis of wide arrays of analytes in biological samples, each demonstrating varying concentrations and physicochemical properties. Metabolomics is known to have strong potential for use in perinatal medicine [12,30]. Previous studies exploited this approach to investigate both physiological [13,31] and pathological [32,33] metabolic changes in AF occurring during pregnancy. We decided to analyse, for the first time, to the best of our knowledge, the pathophysiological mechanisms involved in increased nuchal fold size, which represents a crucial node in the evaluation of pregnancy progression. In our prospective study, we designed a specific experimental metabolomic workflow to assay the concentration of several metabolites from AF in euploid foetuses with enlarged NT, in order to obtain information regarding the underlying mechanisms that may precipitate this condition when other aetiologies are not evident.

Specifically, GC-MS has vast potential as a tool for investigations of this type and, together with multivariate analytical tools, allows the study of both small- and large-scale variations in metabolomic profile, which contribute to the final result.

The amniotic fluid of >NT foetuses showed a distinct metabolic profile compared with the control group. In addition, we found alterations in a number of metabolic pathways involved in the following: synthesis and degradation of ketone bodies, butyrate metabolism, valine, leucine, and isoleucine biosynthesis. Interestingly, the metabolic profile of the amniotic fluid of the enrolled subjects strongly correlated with the parameters NT and CRL, directly carrying information relative to the fetal composition, suggesting a close dependence between the metabolic imprint and the specific intrauterine contest. Weak correlations were evidenced when clinical parameters were considered such as PAPP-A MoM and free β-hCG MoM, which mainly reflect placental activity. Moreover, when we considered the correlations between only the clinical parameters, we observed a strong correlation of the NT values with the CRL, but no correlations were evidenced between the NT values and PAPP-A MoM and free β-hCG MoM, as expected.

Several metabolic modifications occur in a woman’s body during pregnancy, for reasons such as ensuring the appropriate use of energy resources. In this light, ketone bodies are essential substrates used as glucose substitutes to supplement the metabolism of both the mother and the foetus. Indeed, oxidation of ketones is an important contributor to mammalian energy metabolism within extrahepatic tissues in myriad physiological states, including pregnancy, and is a vital alternative metabolic fuel source [34]. It is important to consider that human pregnancy is characterised by alterations in maternal lipid metabolism [35], which is strongly correlated with ketone biosynthesis. More interestingly, maternal ketogenesis allows the foetus to exploit these molecules not only as energy fuels, but also as substrates for brain lipid synthesis [34]. Physiologically, lipolysis increases the availability and use of free fatty acids as energy substrates for the mother’s body in place of glucose, consumed mostly by the foetus [36]. These mechanisms represent the basis of the increased ketogenesis during pregnancy. In contrast, >NT foetuses showed a decreased level of 3-hydroxybutyrate in amniotic fluid.

The transfer of ketone bodies across the placenta occurs via passive diffusion down their concentration gradient or by specific placental carrier-dependent transport [37]. On one hand, unrestricted and rapid arrival of ketone bodies from maternal to foetal circulation guarantees embryonic brain development under conditions of nutrient deficiency [38], though on the other, high levels of these compounds are strongly linked with an increased incidence of foetal abnormalities such as malformations, impaired neurophysiological development, and still birth [39]. Alteration of these pathways is likely strongly correlated with the other altered pathways in the >NT class, including butyrate (butanoate) metabolism. Butanoate metabolism explicates the metabolic destiny of several short chain fatty acids or short chain alcohols normally produced by intestinal fermentation. Many of these molecules are eventually used in the production of ketone bodies; the creation of short-chain lipids; or as precursors to the citrate cycle, glycolysis, or glutamate synthesis.

Cholesterol is another discriminating metabolite that captured our attention in this study; its concentration is significantly diminished in >NT foetuses compared with the control class. It has been estimated that there must be a net accumulation of approximately 1.5–2.0 g of cholesterol for each kg of tissue added to the body of the developing embryo [35,40]. As a consequence of its high cholesterol demand, the developing foetus obtains this sterol either as a product of de novo biosynthesis [41] or from maternally derived deposits in the yolk sac and the placenta [42]. This molecule plays a key role in embryonic and foetal development, with myriad functions. Cholesterol is an essential component of cell membranes, influencing membrane fluidity and passive permeability. Cholesterol is also a precursor for bile acids and steroid hormones (e.g., glucocorticoids that are actively synthesised in foetal adrenal glands, especially during late pregnancy) and has roles in maintaining cellular homeostasis and signalling under a variety of both physiological and pathological states. Moreover, it plays important roles in cell proliferation, differentiation, as well as cell-to-cell communication [35,40]. Low maternal serum cholesterol levels during pregnancy are associated with reduced birth weights, and inversely correlated with the incidence of microcephaly, while maternal gestational hypercholesterolemia promotes early atherogenicity [35].

Valine, leucine, and isoleucine biosynthesis was another altered pathway, strongly correlated with the decrease in threonine availability. Amino acids play a critical role in embryonic development [43]. The human placenta expresses over 15 different amino acid transporters, and each is responsible for the uptake of several different amino acids [44]. Reduced fetal plasma concentrations of several amino acids were also observed in intrauterine growth-restriction (IUGR) at term [45,46]. The transport of amino acid and in particular that of valine, leucine, and isoleucine across the placenta is reportedly impaired in fetal growth restriction [47] and preeclampsia [48]. Interestingly, placental amino acids transporters were found to also be altered in IUGR human foetuses [49]. Indeed, leucine transport was found to be reduced in human term placentas of IUGR pregnancies [50]. According to data in the HMDB [51], branched chain amino acids play a critical role in stress and energy metabolism, which we supposed to be altered in >NT foetuses. Valine is metabolized and directed to carbohydrate synthesis, leucine moves to fat synthesis, and isoleucine contributes to both [52]. Finally, leucine is known to play an important role in fetal nutrition [53,54].

## 5. Conclusions

To summarize, a complex mix of potential altered pathways putative for the presence of >NT in euploid foetuses was identified in this study. Large-scale studies of metabolites are fundamental in understanding cellular metabolism and human pathophysiology and, in this perspective, one of the weak points of this study could be the sample size. Indeed, the number of the fetuses with >NT analysed in this study represents the minimum subjects per group to be considered for pilot studies [23], and it is reasonable to assume that the study should be expanded for its conclusions to be more robust. At the moment, it is not easy to explain what happens during fetal development from the metabolomic point of view, especially regarding this topic. This is because of two main reasons: first of all, there are no studies based on the evaluation of the fetal metabolic profile in euploid foetuses with thickened NT and it is difficult to have a comparison with other results. On the other hand, we miss the serum data of the mothers, which could be extremely important to speculate regarding the biological interpretation of the data. Conversely, it is also important to underline that this study demonstrated that, in AF of euploid foetuses with thickened NT, the metabolic profile was significantly altered compared with control subjects, and this represents a good starting point, for the scientific community, to offer a promising predictive model of what could happen during the late phases of pregnancy or after birth (e.g., intrauterine growth restriction, small for gestational age, extremely low birth weight, and so on). Finally, this approach may contribute to improving the understanding of some pathological mechanisms that represent a focal point in the evaluation of pregnancy during the first trimester.

## Figures and Tables

**Table 1:** Demographic and clinical characteristics of the enrolled patients.

Classes	N	Age (Range)	Ultrasound		Biochemical Assay	
			NT ± SD (mm)	CRL± SD (mm)	PAPP-A MoM ± SD (UI/L)	Free β-hCG MoM ± SD (mlU/mL)
Controls	27	35.1 (23–44)	1.41 ± 0.17	54.4 ± 4.3	0.77 ± 0.28	1.43 ± 1
Enlarge Nuchal Translucency	12	37.2 (29–44)	2.5 ± 0.3	62.1 ± 7.5	0.97 ± 0.47	1.93 ± 1.7

NT = nuchal translucency; CRL = crown-rump length of the fetus; PAPP-A MoM = pregnancy associated plasma protein A; free β-hCG MoM = beta-subunit of human chorionic gonadotropin.

**Table 2:** Statistical parameters of the discriminating metabolites for the separation between C and >NT patients.

Metabolites	Trend >NT vs. C	*p*-Value	Corrected *p*-Value	ROC Curve			
				Area	Std. Error	Confidence Interval	*p*-Value
2-OH-Butyrate	↓	0.007	0.02	0.77	0.07	0.62–0.92	0.008
3-OH-Butyrate	↓	0.0006	0.004	0.83	0.06	0.7–0.96	0.001
1,5 Anydro Sorbitol	↑	0.02	0.03	0.73	0.08	0.56–0.90	0.02
Cholesterol	↓	0.0009	0.004	0.82	0.06	0.7–0.95	0.001
Erithronic acid	↓	0.007	0.02	0.76	0.08	0.6–0.9	0.008
Fructose	↓	0.003	0.01	0.79	0.07	0.65–0.94	0.003
Malic Acid	↓	0.0003	0.001	0.85	0.06	0.7–0.97	0.0005
Threitol	↓	0.0002	0.001	0.86	0.05	0.74–0.97	0.0004
Threonine	↓	0.0008	0.004	0.83	0.06	0.7–0.95	0.001

## Data Availability

The data presented in this study are available on request from the corresponding author.

## Supplementary Materials

The following are available online at https://www.mdpi.com/article/10.3390/life11090913/s1, Figure S1: Graphical comparisons of the demographic and clinical characteristics of the control subjects and >NT foetuses. *** = *p* = 0.0001; **** = *p* < 0.0001 after Student’s *t*-test. Figure S2: Unsupervised PCA model of the foetuses with enlarged nuchal translucency (black circles) and controls (white boxes).

## References

[1] Ferguson-Smith M.A., Bianchi D.W. (2010). Prenatal Diagnosis: Past, present, and future. Prenat. Diagn..

[2] Norton M.E., Kuller J.A., Dugoff L. (2019). Perinatal Genetics.

[3] Norton M.E., Jacobsson B., Swamy G.K., Laurent L.C., Ranzini A.C., Brar H., Tomlinson M.K., Pereira L., Spitz J.L., Hollemon D. (2015). Cell-free DNA Analysis for Noninvasive Examination of Trisomy. N. Engl. J. Med..

[4] Monni G., Zoppi M.A., Iuculano A., Piras A., Arras M. (2014). Invasive or non-invasive prenatal genetic diagnosis?. J. Perinat. Med..

[5] Levy B., Stosic M., Levy B. (2019). Traditional Prenatal Diagnosis: Past to Present. Prenatal Diagnosis.

[6] Nicolaides K.H., Brizot M.L., Snijders R.J.M. (1994). Fetal nuchal translucency: Ultrasound screening for fetal trisomy in the first trimester of pregnancy. BJOG Int. J. Obstet. Gynaecol..

[7] Nafziger E., Vilensky J.A. (2014). The anatomy of nuchal translucency at 10–14 weeks gestation in fetuses with trisomy 21: An incredible medical mystery. Clin. Anat..

[8] Zoppi M.A., Ibba R.M., Floris M., Manca F., Axiana C., Monni G. (2003). Changes in nuchal translucency thickness in normal and abnormal karyotype fetuses. BJOG Int. J. Obstet. Gynaecol..

[9] Kagan K.O., Wright D., Spencer K., Molina F.S., Nicolaides K.H. (2008). First-trimester screening for trisomy 21 by free beta-human chorionic gonadotropin and pregnancy-associated plasma protein-A: Impact of maternal and pregnancy characteristics. Ultrasound Obstet. Gynecol..

[10] Souka A.P., Von Kaisenberg C.S., Hyett J.A., Sonek J.D., Nicolaides K.H. (2005). Increased nuchal translucency with normal karyotype. Am. J. Obstet. Gynecol..

[11] Syngelaki A., Hammami A., Bower S., Zidere V., Akolekar R., Nicolaides K.H. (2019). Diagnosis of fetal non-chromosomal abnormalities on routine ultrasound examination at 11–13 weeks’ gestation. Ultrasound Obstet. Gynecol..

[12] Monni G., Atzori L., Corda V., Dessolis F., Iuculano A., Hurt K.J., Murgia F. (2021). Metabolomics in prenatal medicine: A review. Front. Med. (Lausanne).

[13] Orczyk-Pawilowicz M., Jawien E., Deja S., Hirnle L., Zabek A., Mlynarz P. (2016). Metabolomics of Human Amniotic Fluid and Maternal Plasma during Normal Pregnancy. PLoS ONE.

[14] Monni G., Murgia F., Corda V., Peddes C., Iuculano A., Tronci L., Balsamo A., Atzori L. (2019). Metabolomic Investigation of β-Thalassemia in Chorionic Villi Samples. J. Clin. Med..

[15] Murgia F., Iuculano A., Peddes C., Santoru M.L., Tronci L., Deiana M., Atzori L., Monni G. (2019). Metabolic fingerprinting of chorionic villous samples in normal pregnancy and chromosomal disorders. Prenat. Diagn..

[16] Menon R., Jones J., Gunst P.R., Kacerovsky M., Fortunato S.J., Saade G.R., Basraon S. (2014). Amniotic Fluid Metabolomic Analysis in Spontaneous Preterm Birth. Reprod. Sci..

[17] Iuculano A., Murgia F., Peddes C., Santoru M.L., Tronci L., Deiana M., Balsamo A., Euser A., Atzori L., Monni G. (2019). Metabolic characterization of amniotic fluids of fetuses with enlarged nuchal translucency. J. Perinat. Med..

[18] Dettmer K., Aronov P.A., Hammock B.D. (2007). Mass spectrometry-based metabolomics. Mass Spectrom. Rev..

[19] Fiehn O. (2016). Metabolomics by Gas Chromatography—Mass Spectrometry: Combined Targeted and Untargeted Profiling. Curr. Protoc. Mol. Biol..

[20] Ellis D.I., Dunn W.B., Griffin J.L., Allwood J.W., Goodacre R. (2007). Metabolic fingerprinting as a diagnostic tool. Pharmacogenomics.

[21] Poddighe S., Murgia F., Lorefice L., Liggi S., Cocco E., Marrosu M.G., Atzori L. (2017). Metabolomic analysis identifies altered metabolic pathways in Multiple Sclerosis. Int. J. Biochem. Cell Biol..

[22] Weckwerth W., Morgenthal K. (2005). Metabolomics: From pattern recognition to biological interpretation. Drug Discov. Today.

[23] Julious S.A. (2005). Sample size of 12 per group rule of thumb for a pilot study. Pharmaceut. Stat..

[24] Murgia F., Atzori L., Carboni E., Santoru M.L., Hendren A., Pisanu A., Caboni P., Boi L., Fusco G., Carta A.R. (2020). Metabolomics Fingerprint Induced by the Intranigral Inoculation of Exogenous Human Alpha-Synuclein Oligomers in a Rat Model of Parkinson’s Disease. Int. J. Mol. Sci..

[25] Eriksson L., Byrne T., Johansson E., Trygg J., Vikström C. (2013). Multi- and Megavariate Data Analysis Basic Principles and Applications. Umetrics Acad..

[26] Trygg J., Holmes E., Lundstedt T. (2007). Chemometrics in metabonomics. J. Proteome Res..

[27] Lindgren F., Hansen B., Karcher W., Sjöström M., Eriksson L. (1996). Model validation by permutation tests: Applications to variable selection. J. Chemom..

[28] Wold S., Sjöström M., Eriksson L. (2001). PLS-regression: A basic tool of chemometrics. Chemom. Intell. Lab. Syst..

[29] Chong J., Wishart D.S., Xia J. (2019). Using MetaboAnalyst 4.0 for Comprehensive and Integrative Metabolomics Data Analysis. Curr. Protoc. Bioinform..

[30] Zhang A., Sun H., Yan G., Wang P., Wang X. (2015). Metabolomics for Biomarker Discovery: Moving to the Clinic. BioMed Res. Int..

[31] Cohn B.R., Joe B.N., Zhao S., Kornak J., Zhang V.Y., Iman R., Kurhanewicz J., Vahidi K., Yu J., Caughey A.B. (2009). Quantitative metabolic profiles of 2nd and 3rd trimester human amniotic fluid using (1) H HR-MAS spectroscopy. MAGMA.

[32] Graca G., Duarte I.F., Barros A.S., Goodfellow B.J., Diaz S.D.O., Pinto J.I.M., Carreira I.M., Galhano E., Pita C., Gil A.M. (2010). Impact of prenatal disorders on the metabolic profile of second trimester amniotic fluid: A nuclear magnetic resonance metabonomic study. J. Proteome Res..

[33] Graça G., Goodfellow B.J., Barros A.S., Diaz S., Duarte I.F., Spagou K., Veselkov K., Want E.J., Lindon J.C., Carreira I.M. (2012). UPLC-MS metabolic profiling of second trimester amniotic fluid and maternal urine and comparison with NMR spectral profiling for the identification of pregnancy disorder biomarkers. Mol. Biosyst..

[34] Puchalska P., Crawford P.A. (2017). Multi-dimensional Roles of Ketone Bodies in Fuel Metabolism, Signaling, and Therapeutics. Cell Metab..

[35] Zeng Z., Liu F., Li S. (2017). Metabolic Adaptations in Pregnancy: A Review. Ann. Nutr. Metab..

[36] Frise C.J., Mackillop L., Joash K., Williamson C. (2013). Starvation ketoacidosis in pregnancy. Eur. J. Obstet. Gynecol. Reprod. Biol..

[37] Alonso de la Torre S.R., Serrano M.A., Medina J.M. (1992). Carrier-mediated beta-D-hydroxybutyrate transport in brush-border membrane vesicles from rat placenta. Pediatr. Res..

[38] Ville D., Chiron C., Laschet J., Dulac O. (2015). The ketogenic diet can be used successfully in combination with corticosteroids for epileptic encephalopathies. Epilepsy Behav..

[39] Tatone E.H., Duffield T.F., Capel M.B., DeVries T.J., LeBlanc S.J., Gordon J.L. (2016). A randomized controlled trial of dexamethasone as an adjunctive therapy to propylene glycol for treatment of hyperketonemia in postpartum dairy cattle. J. Dairy Sci..

[40] Dietschy J.M., Turley S.D., Spady D.K. (1993). Role of liver in the maintenance of cholesterol and low density lipoprotein homeostasis in different animal species, including humans. J. Lipid Res..

[41] Herrera E. (2002). Lipid metabolism in pregnancy and its consequences in the fetus and newborn. Endocrine.

[42] Woollett L.A., Heubi J.E., Feingold K.R., Anawalt B., Boyce A., Chrousos V., de Herder W.W., Dhatariya K., Dungan K., Ashley Grossman A., Hershman J.M., Hofland V. (2000). Fetal and Neonatal Cholesterol Metabolism.

[43] Cetin I., Marconi A.M., Corbetta C., Lanfranchi A., Baggiani A., Battaglia F., Pardi G. (1992). Fetal amino acids in normal pregnancies and in pregnancies complicated by intrauterine growth retardation. Early Hum. Dev..

[44] Jansson T. (2001). Amino acid transporters in the human placenta. Pediatr. Res..

[45] Cetin I., Marconi A.M., Bozzetti P., Sereni L.P., Corbetta C., Pardi G., Battaglia F.C. (1988). Umbilical amino-acid concentrations in appropriate and small for gestational-age infants: A biochemical difference present in utero. Am. J. Obstet. Gynecol..

[46] Pantham P., Rosario F.J., Weintraub S.T., Nathanielsz P., Powell T., Li C., Jansson T. (2016). Down-Regulation of Placental Transport of Amino Acids Precedes the Development of Intrauterine Growth Restriction in Maternal Nutrient Restricted Baboons. Biol. Reprod..

[47] Paolini C.L., Marconi A.M., Ronzoni S., Di Noio M., Fennessey P.V., Pardi G., Battaglia F.C. (2001). Placental transport of leucine, phenylalanine, glycine, and proline in intrauterine growth-restricted pregnancies. J. Clin. Endocrinol. Metab..

[48] Lisonkova S., Joseph K.S. (2013). Incidence of preeclampsia: Risk factors and outcomes associated with early- versus late-onset disease. Am. J. Obstet. Gynecol..

[49] Mahendran D., Donnai P., Glazier J.D., D’Souza S.W., Boyd R.D., Sibley C.P. (1993). Amino acid (system A) transporter activity in microvillous membrane vesicles from the placentas of appropriate and small for gestational age babies. Pediatr. Res..

[50] Jansson T., Scholtbach V., Powell T.L. (1998). Placental transport of leucine and lysine is reduced in intrauterine growth restriction. Pediatr. Res..

[51] Wishart D.S., Jewison T., Guo A.C., Wilson M., Knox C., Liu Y., Djoumbou Y., Mandal R., Aziat F., Dong E. (2013). HMDB 3.0—The Human Metabolome Database in 2013. Nucleic Acids Res..

[52] Bahado-Singh R.O., Syngelaki A., Mandal R., Graham S.F., Akolekar R., Han B., Bjondahl T.C., Dong E., Bauer S., Alpay-Savasan Z. (2017). Metabolomic determination of pathogenesis of late-onset preeclampsia. J. Matern.-Fetal Neonatal Med..

[53] Battaglia F.C., Regnault T.R.H. (2001). Placental Transport and Metabolism of Amino Acids. Placenta.

[54] Cetin I. (2001). Amino acid interconversions in the fetal-placental unit: The animal model and human studies in vivo. Pediatr. Res..

