# Supplementary material for: Metabolomics Analysis of Amniotic Fluid in Euploid Foetuses with Thickened Nuchal Translucency by Gas Chromatography-Mass Spectrometry
Source: Life (Basel). 2021 Sep 2;11(9):913. doi: 10.3390/life11090913 (PMC8466859; doi:10.3390/life11090913)
Supplement: Supplementary file 1 [file life-11-00913-s001.zip › life-1293292-supplementary.pdf]

Supplementary Materials

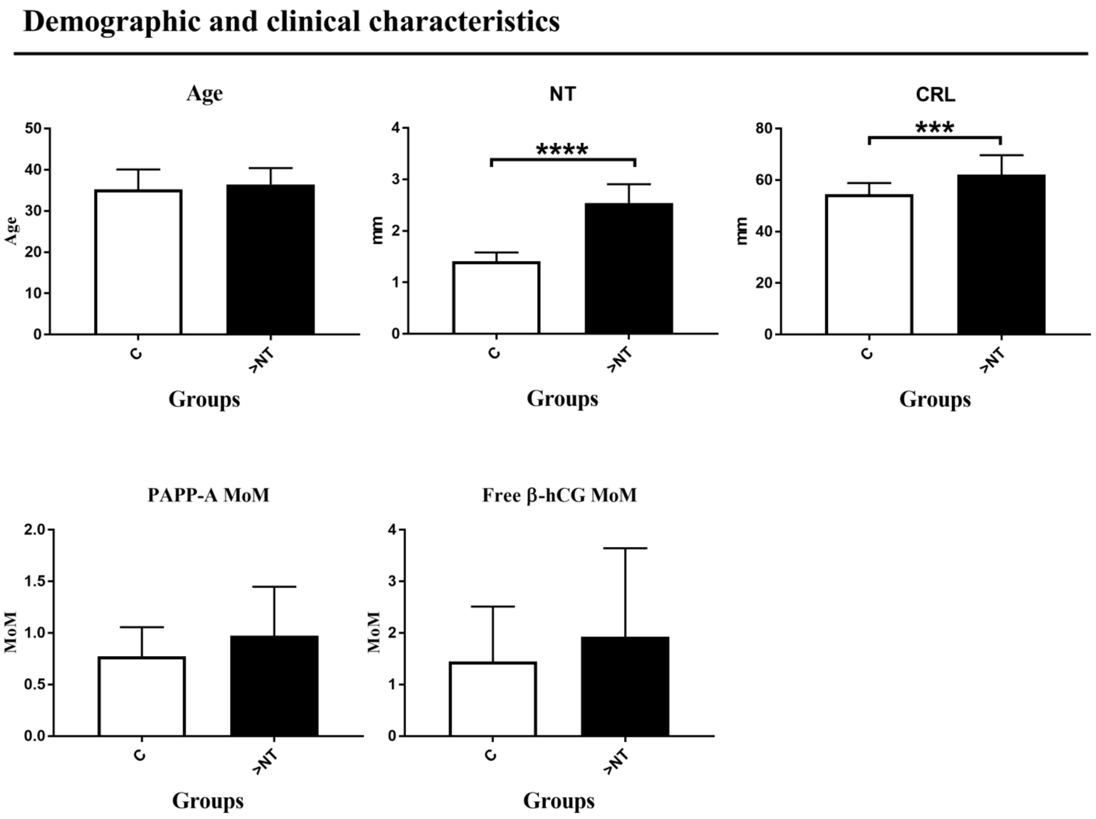

Figure S1. Graphical comparisons of the demographic and clinical characteristics of the control subjects and >NT foetuses. *** = $p = 0.0001$; **** = $p < 0.0001$ after Student's t-test.

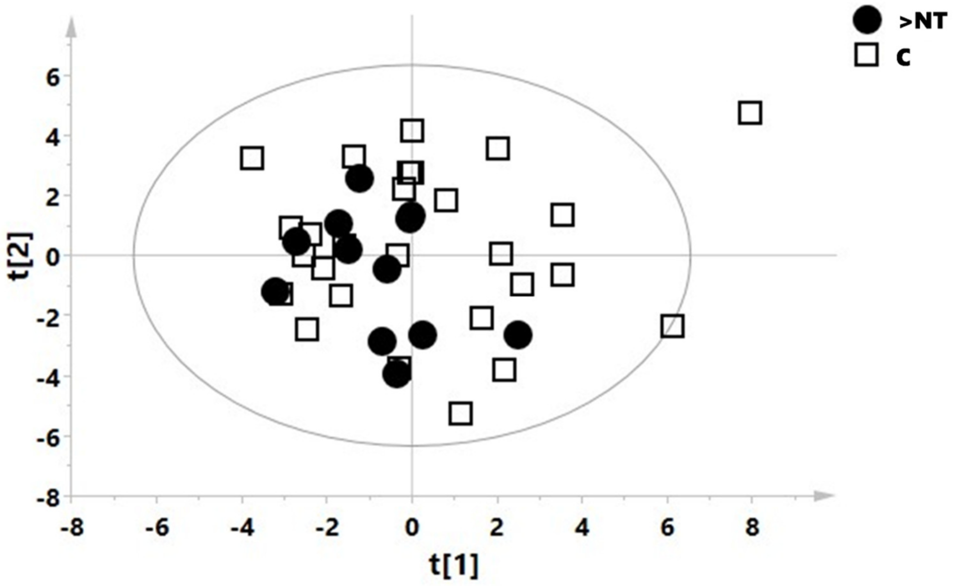

Figure S2. Unsupervised PCA model of the foetuses with enlarged nuchal translucency (black circles) and controls (white boxes).
